# Surgical Management of Gallstone Ileus: Laparoscopic and Open Approaches in a Single-Center Experience

**DOI:** 10.3390/medicina61122174

**Published:** 2025-12-07

**Authors:** Sangar Abdullah, Güney Özkaya, Adnan Gündoğdu, Başar Can Turgut, Sefa Ergün

**Affiliations:** 1 Department of General Surgery, Sancaktepe Şehit Prof. Dr. Ilhan Varank Training and Research Hospital, Istanbul 34785, Türkiye; 2 Department of General Surgery, Istanbul Training and Research Hospital, Istanbul 34400, Türkiye; 3 Department of General Surgery, Cerrahpasa Cerrahpasa Medical Faculty, Istanbul University, Istanbul 34452, Türkiye

**Keywords:** gallstone ileus, bilioenteric fistula, Rigler’s triad, laparoscopy

## Abstract

*Background and Objectives*: Gallstone ileus (GI) is a rare mechanical intestinal obstruction resulting from gallstone impaction through a bilioenteric fistula, accounting for 1–4% of mechanical intestinal obstructions. This study aimed to evaluate the clinical approach, surgical management, and outcomes in a cohort of surgically treated GI patients. *Materials and Methods*: A retrospective cohort analysis of 12 patients diagnosed with GI and treated surgically between January 2018 and December 2024 was conducted. Data collected included demographics, clinical presentation, imaging findings, surgical approach, and postoperative outcomes. Descriptive statistics were used due to small sample size. *Results*: All patients presented with abdominal pain and vomiting, with delayed admission (mean 3.83 ± 2.08 days). Rigler’s triad was identified on CT in 91.7% of cases. The distal ileum (66.7%) was the most common site of obstruction, with a mean stone size of 3.19 ± 0.6 cm. Surgical management included enterolithotomy alone (66.7%, n = 8) and one-stage procedures (33.3%, n = 4). Laparoscopic approaches were employed successfully in 41.7% of cases (n = 5) without the need for conversion. Postoperative complications occurred in 41.7% of patients, with 8.3% mortality (n = 1). One recurrence was observed during a median (IQR) follow-up period of 33.00 (7.00–66.00) months. *Conclusions*: GI management should be individualized based on patient risk factors. In our cohort, enterolithotomy alone was the most common approach for high-risk elderly patients, while one-stage procedures were performed in stable patients with residual gallstones. Laparoscopic approaches were utilized in selected hemodynamically stable patients with appropriate surgical expertise. Our experience suggests that minimally invasive techniques can be successfully achieved in this traditionally challenging condition with careful patient selection.

## 1. Introduction

Gallstone ileus (GI) is a rare mechanical intestinal obstruction caused by impaction of a gallstone within the gastrointestinal tract, accounting for approximately 1–4% of all mechanical intestinal obstructions, but up to 25% in patients aged over 70 years [1,2,3]. The condition predominantly affects older women with multiple comorbidities and occurs 3.5 times more frequently in women than in men. These factors contribute to high postoperative morbidity and mortality rates ranging from 7% to 27% [3].

GI typically develops when a gallstone migrates through a bilioenteric fistula (BEF), most commonly a cholecystoduodenal fistula, and becomes impacted in the intestinal lumen. The terminal ileum is the most common site of obstruction [4,5].

The clinical presentation is often nonspecific and depends on the site of obstruction, making diagnosis challenging. Contrast-enhanced computed tomography (CT) is the gold-standard diagnostic tool, with a sensitivity of 90–93% and the ability to identify the classic Rigler’s triad: pneumobilia, ectopic gallstone, and intestinal obstruction [6,7].

Treatment primarily involves surgical stone extraction. Enterolithotomy alone is the most commonly performed procedure in this elderly, high-risk population. Although open surgery has been the traditional approach, laparoscopic techniques have been increasingly described in recent years [8,9]. However, current evidence is limited to small case series, and the feasibility, safety, and appropriate indications for laparoscopy in GI remain insufficiently defined. There is no clear consensus regarding the surgical approach (laparoscopic versus open) or the extent of surgery (enterolithotomy alone versus one-stage procedure including cholecystectomy and fistula repair), nor are the clinical factors that predict optimal management strategies clear.

This study aims to address this knowledge gap by evaluating our institutional seven-year experience with GI, focusing on the laparoscopic utilization rate, factors influencing surgical technique selection, and long-term outcomes.

## 2. Materials and Methods

### 2.1. Study Design and Ethical Approval

This study was designed as a retrospective cohort analysis at Istanbul Sancaktepe Şehit Prof. Dr. Ilhan Varank Training and Research Hospital, Turkey. This study was prepared following the STROBE (Strengthening the Reporting of Observational Studies in Epidemiology) guidelines for reporting observational studies. Ethical approval was obtained from the hospital’s Scientific Research Ethics Committee (Approval No: E-46059653-050.04-282350113, Date: 18 July 2025). In accordance with institutional policy, the ethics committee permits retrospective approval for studies that utilize existing clinical records. The study adhered to the principles of the Declaration of Helsinki.

### 2.2. Patient Selection

**Figure 1:**
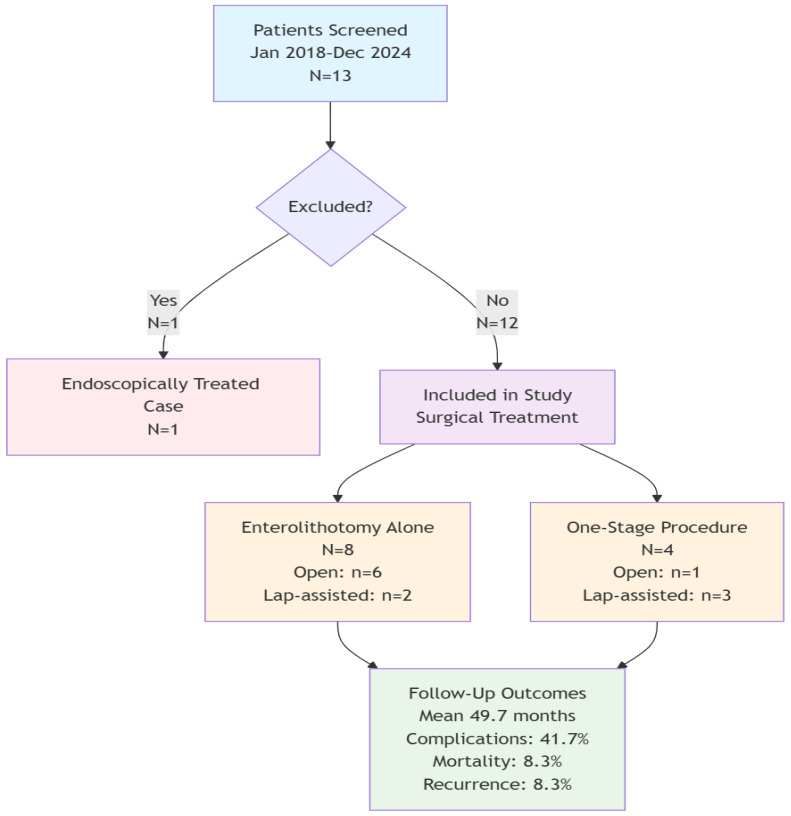
Patient selection flowchart showing study inclusion and exclusion criteria.

Between January 2018 and December 2024, 13 patients were diagnosed with GI at our institution (Figure 1). The study included 12 patients who met the following inclusion criteria: (1) confirmed diagnosis of GI based on clinical, radiological, and/or intraoperative findings; (2) surgical management; (3) age 18 years or older; (4) at least 6 months of follow-up. One patient who was treated endoscopically was excluded from the study.

### 2.3. Data Collection

Data were retrospectively extracted from the hospital’s electronic medical records and operative notes, with data accuracy confirmed by cross-referencing imaging reports, laboratory results, and follow-up documentation. Collected variables included the following:

Demographics: Age, sex, American Society of Anesthesiologists (ASA) score, and the Charlson Comorbidity Index (CCI). The CCI was calculated to provide objective comorbidity risk stratification.

Clinical Presentation: Comorbidities, signs and symptoms at the time of admission, time from symptom onset to admission, and history of acute cholecystitis or cholelithiasis.

Diagnostics: Imaging findings included CT-scans, ultrasonography, and plain X-rays, laboratory values included white blood cell count (WBC), hemoglobin (Hb), C-reactive protein (CRP), aspartate aminotransferase (AST), alanine aminotransferase (ALT), gamma-glutamyl transferase (GGT), lactate dehydrogenase (LDH), total bilirubin (T. Bil), direct bilirubin (D. Bil), indirect bilirubin (Ind bil), urea, potassium (K), calcium (Ca), sodium (Na), and creatinine.

Operative details: Type of procedure (e.g., open or laparoscopic enterolithotomy alone, open or laparoscopic one-stage procedure enterolithotomy + cholecystectomy + fistula repair), operation time (in minutes), intraoperative findings (bilioenteric fistula site, number and size of stones).

Postoperative and follow-up outcomes: Postoperative complications (classified by Clavien-Dindo system), length of hospital stay (days), intensive care unit (ICU) stay (days), follow-up period (month), and recurrence or mortality.

### 2.4. Surgical Approaches

Before admission, all patients had blood tests and underwent plain X-ray and contrast-enhanced CT at the emergency department of our hospital. After admission, the patients were put on nil by mouth, resuscitated with intravenous electrolyte and fluid replacement, and had a nasogastric tube inserted for decompression. All patients received broad-spectrum intravenous antibiotic prophylaxis (third-generation cephalosporin combined with metronidazole) within one hour before surgery and discontinued it after 24 h unless there was an indication for continuation. Postoperative deep vein thrombosis (DVT) prophylaxis with low-molecular-weight heparin was administered unless contraindicated.

Stable patients were observed for 2–3 days before a decision on surgery was made; those with signs of an acute abdomen were operated on immediately.

#### Surgical Approach Selection

Choice between enterolithotomy and one-stage procedure:

The choice between enterolithotomy and one-stage procedure was individualized according to each patient’s risk stratification and clinical factors. Enterolithotomy alone was generally favored in patients with: (1) ASA IV classification; (2) advanced age or low physiological reserve; (3) hemodynamic instability or inability to tolerate prolonged anesthesia; (4) absence of residual gallstones in the gallbladder on preoperative CT; and (5) the presence of dense adhesions or severe inflammation intraoperatively.

Patients with: (1) ASA II-III classification; (2) younger age with better functional status; (3) hemodynamic stability with ability to tolerate extended operative time; (4) residual gallstones in the gallbladder on preoperative CT; and (5) favorable intraoperative findings, including limited inflammation and accessible fistula anatomy, were considered to receive one-stage procedures.

Choice between laparoscopic and open approach:

Laparoscopic approach was favored when the following criteria were met: (1) ASA II-III classification (no laparoscopy was performed in ASA IV patients); (2) hemodynamic stability; (3) absence of massive bowel distension on preoperative CT; (4) absence of suspected perforation or generalized peritonitis; (5) presence of experienced laparoscopic surgeon and team members; and (6) intraoperative feasibility assessment including adequate visualization and safe bowel manipulation.

Acknowledgment of Selection Bias:

The selection criteria were not protocolized; instead, the decisions were made individually by the surgical team based on clinical judgment. Multiple factors were considered, including patient condition, radiological findings, intraoperative assessment, institutional resources, and surgeon expertise. While this reflects real-world clinical practice, it introduces selection bias. The non-randomized treatment allocation precludes direct statistical comparison of outcomes between surgical approaches and limits the generalizability of our findings.

**Figure 2:**
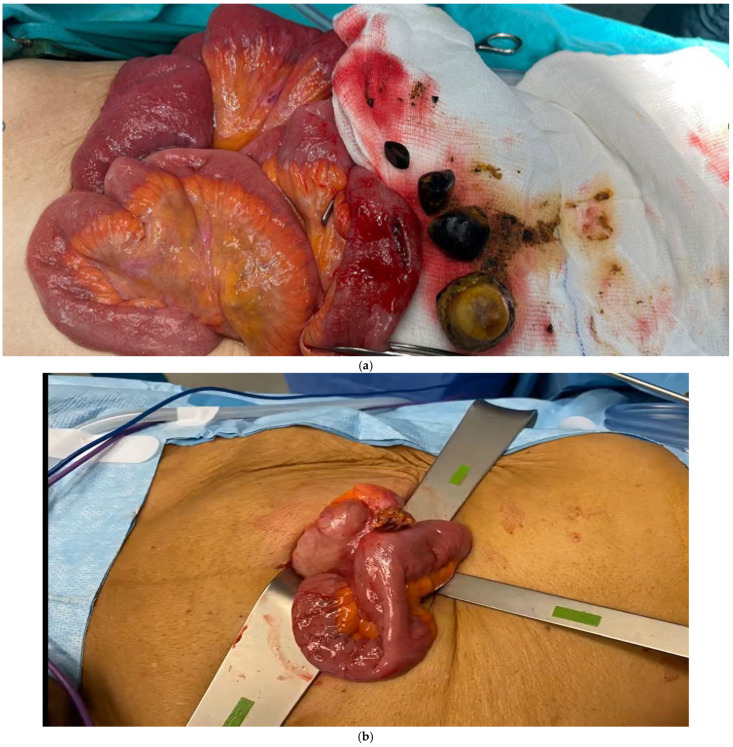

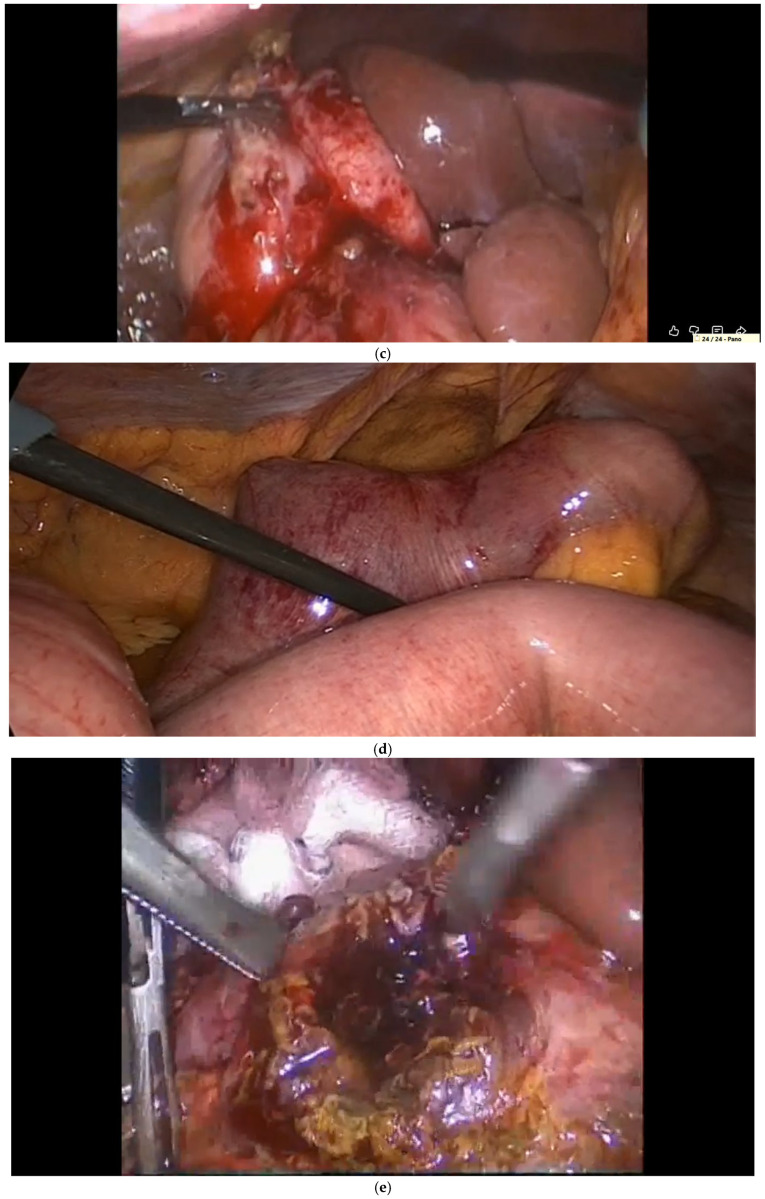
Operative images; (**a**) open enterolithotomy. (**b**) laparoscopic-assisted enterolithotomy. (**c**) cholecystodudenal fistula. (**d**) distal ileum stone impaction. (**e**) duodenal fistula orifice.

All procedures were carried out under general anesthesia. For patients undergoing open enterolithotomy, a midline incision above and below the umbilicus was made, followed by enterotomy and stone extraction, after which the enterotomy site was closed with continuous or interrupted 3/0 Vicryl sutures (Figure 2a). All laparoscopic procedures utilized a laparoscopy-assisted technique. In the laparoscopic enterolithotomy, after pneumoperitoneum and exploration, the stone impaction site was identified and through a small 4–5 cm incision in the umbilicus, the small bowel was exteriorized, and the same procedure as the open approach was carried out (Figure 2b). For patients undergoing the open one-stage procedure, enterolithotomy was performed, followed by cholecystectomy and fistula repair, while in the laparoscopic one-stage group, cholecystectomy and fistula repair were performed laparoscopically, followed by bowel exteriorization through a small umbilical incision for enterolithotomy. The enterotomy and fistula sites were closed with 3-0 Vicryl sutures, and a surgical drain was placed in all patients (Figure 2c–e). If a patient who was planned for a one-stage procedure deteriorated during surgery, only enterolithotomy was performed.

### 2.5. Statistical Analysis

SPSS (Statistical Package for the Social Sciences) 25.0 software (IBM Corp., Armonk, NY, USA) program was used for statistical analysis of data. Categorical measurements were summarized as numbers and percentages (using the n/N format for explicit reporting of counts and denominators). Continuous measurements, including all laboratory parameters and time-related variables (e.g., Hospital Stay, Time before Admission, CCI score), were summarized as median and interquartile range (IQR) with full range (Minimum–Maximum) due to the small sample size and expected non-normal distribution. Other continuous measurements (e.g., Age, Stone Size) were summarized as mean ± standard deviation (SD) and range. The Shapiro–Wilk test was used to determine whether the parameters in the study followed a normal distribution.

## 3. Results

During the study period, 12 patients diagnosed with and treated for gallstone ileus were included. Demographic and clinical characteristics are summarized in Table 1, imaging and preoperative findings in Table 2, and surgical details and outcomes in Table 3.

### 3.1. Demographic and Clinical Features

A total of 12 patients were included in the study; the majority were female (83.3%, n = 10) with a mean age of 70.25 ± 10.20 (Table 1). The preoperative ASA score was as follows: ASA II (n = 3), ASA III (n = 6), and ASA IV (n = 3). The median (IQR) CCI was 3.00 (2.00–4.00), objectively quantifying the moderate-to-high comorbidity burden in this cohort. Hypertension (n = 9, 75.0%) and diabetes mellitus (n = 4, 33.3%) were the most common comorbidities, while 16.7% (n = 2) of patients had no comorbidities. All patients presented with abdominal pain (n = 12, 100%), vomiting was frequent (n = 11, 91.7%), and constipation was reported in 16.7% (n = 2) of cases, while fever was noted in only one (n = 1, 8.3%) case. Only two patients (16.7%) had a history of cholelithiasis, and none reported prior acute cholecystitis. The mean time from symptom onset to hospital admission was 3.83 ± 2.08 days. Laboratory analysis revealed elevated inflammatory markers consistent with acute obstruction. The mean C-reactive protein and white blood cell counts were 73.58 ± 80.57 mg/L and 13.99 ± 4.56 × 10^3^/µL respectively.

### 3.2. Imaging and Preoperative Findings

**Figure 3:**
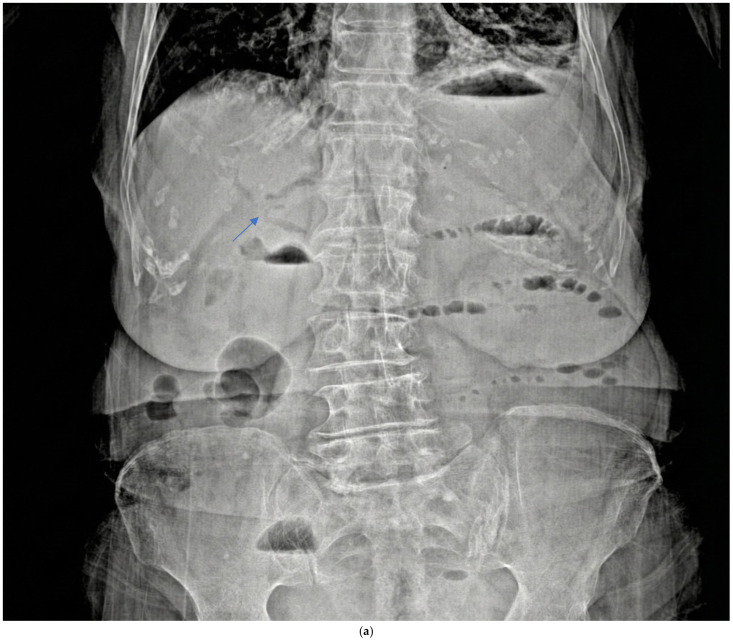

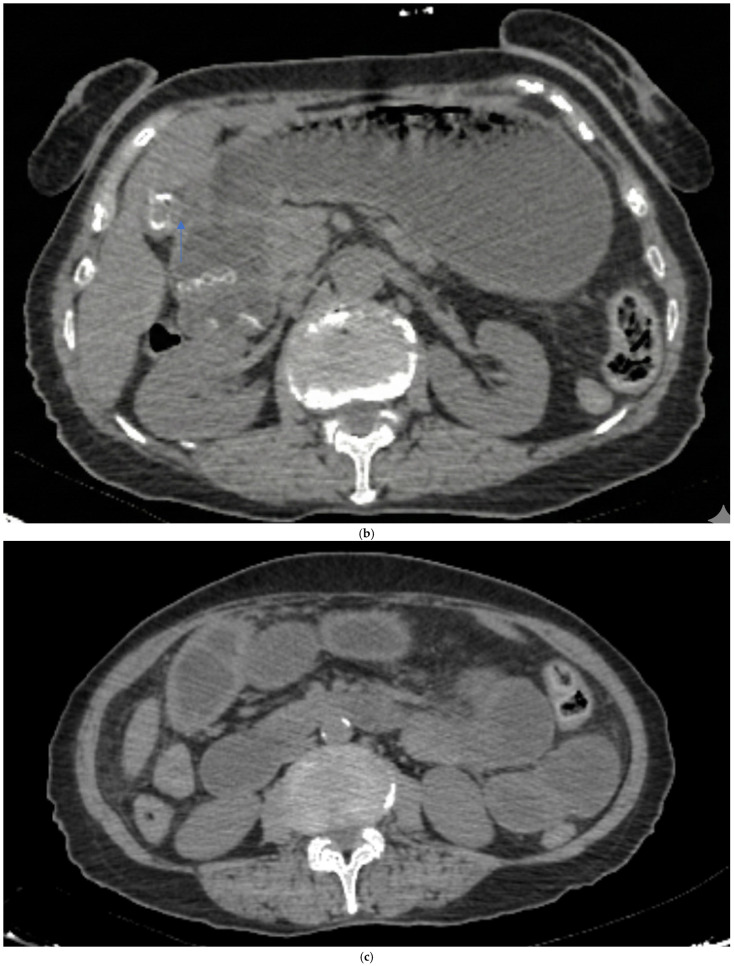

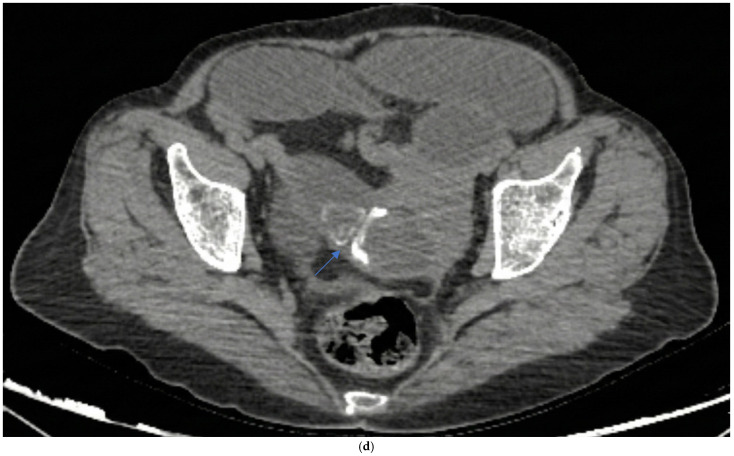
Radiologic images (**a**) plain X-ray air fluid level and pneumobilia (arrow). (**b**) Ct-scan bilioenteric fistula and stone (arrow). (**c**) ileus. (**d**) terminal ileum gallstone (arrow).

Preoperative imaging confirmed GI in all patients (Table 2). All patients had CT and plain X-ray, but none had a preoperative USG in the emergency department. CT revealed a bilioenteric fistula in all cases (12/12) (Figure 3b) and Rigler’s triad in 11/12 patients (91.7%). Air-fluid level and small bowel dilatation > 3 cm were seen in 11/12 patients (91.7%) (Figure 3c); one patient had stone impaction in the duodenum and did not show an air-fluid level. Free fluid was present in 4/12 patients (33.3%), suggesting potential peritonitis. The mean number of stones was 1.6 ± 1.2 (range 1–4), with a mean stone size of 3.19 ± 0.6 cm (range 2.6–5). The stones were primarily located in the distal ileum in 8/12 patients (66.7%) (Figure 3d). Plain X-ray showed an air-fluid level in 11/12 patients (91.7%) and pneumobilia in 2/12 patients (16.7%) (Figure 3a). Previous abdominal surgeries were found in 5/12 patients (41.67%).

### 3.3. Surgical Details and Outcomes

Surgical interventions included enterolithotomy alone or one-stage procedure, with both procedures performed via either laparoscopic or open approaches. Enterolithotomy alone was the most commonly performed procedure in 66.7% (n = 8) of cases, with 75% performed via open approach and 25% via laparoscopic approaches. One-stage procedures were performed in 33.3% (n = 4) of patients, using 3 laparoscopic and 1 open approach. Overall, open approaches were used in 58.3% of cases while laparoscopic approaches were used in 41.7% (Table 3).

Given the small sample size and non-randomized treatment allocation based on patient factors, surgical complexity, and surgeon expertise, formal statistical comparisons between approaches were not performed. Instead, outcomes are presented descriptively for each surgical strategy.

The mean operation time for enterolithotomy alone was 76.82 ± 9.69 min (open: 72.5 ± 6.65 min, n = 6, laparoscopic: 88 ± 2.82 min, n = 2). One-stage procedures had a mean operation time of 150 ± 27.57 min (open: 115 min, n = 1, laparoscopic: 161 ± 18.01 min, n = 3). The site of bilioenteric fistula was bilioduodenal in all cases and no intraoperative complications were seen. A surgical drain was placed in all 12 patients (100%). The median (IQR) hospital stay was 6.00 (5.25–7.75) days. Four patients (33.3%) required postoperative ICU admission, with a mean ICU stay of 1.33 ± 2.57 days; only one patient (8.3%) required mechanical ventilation. According to the Clavien-Dindo classification [10] 58.3% of patients (n = 7) had grade 0 complications, 2 patients (16.7%) had Grade I (wound infection), 2 patients (16.7%) had Grade IIIa complications (one wound dehiscence and one intra-abdominal abscess requiring percutaneous drainage), and 1 patient (8.3%) had grade V complication (mortality). The mortality occurred in a patient who underwent open enterolithotomy and developed aspiration pneumonia on postoperative day 4. The median (IQR) follow-up period was 33.00 (7.00–66.00) months, with recurrence occurring in one patient who underwent a one-stage surgery at another hospital one year after the primary surgery.

## 4. Discussion

Gallstone ileus remains a rare but clinically significant complication of cholelithiasis, constituting 1–4% of all mechanical intestinal obstructions [3,5]. The incidence is higher among the elderly (>70 years), with a female predominance (female-to-male ratio 4:1) [2,3]. Our retrospective cohort study of 12 patients treated surgically for GI over a seven-year period provides valuable insights into the clinical presentation, diagnostic approach, surgical management, and outcomes of this challenging condition at a tertiary care hospital.

In our study, we observed a marked female predominance (83.3%, n = 10) with a mean age of 70.25 ± 10.20 years, consistent with the literature. Notably, our youngest patient was 42 years old, illustrating that while uncommon, GI can occur in younger individuals.

GI occurs as a result of a bilioenteric fistula, which is due to chronic inflammation and ischemia caused by gallstones. The fistula is bilioduodenal in the majority of cases (75–83%) [4], though other sites, including the stomach, small intestine, and colon, have been reported. In our series, all identified fistulas were bilioduodenal (100%). GI occurs when the stone blocks the intestinal lumen. Most stones causing impaction are usually 2–2.5 cm or larger [10]. In our cohort, the mean stone size was 3.19 ± 0.62 cm (range 2.6–5 cm), consistent with the threshold.

The patients in our study were typically elderly with multiple comorbidities, as reflected by their ASA scores (ASA II: n = 3, ASA III: n = 6, ASA IV: n = 3), which contributes to the high postoperative morbidity and mortality rates associated with this condition [3]. This high-risk profile was further quantified by the median (IQR) CCI of 3.00 (2.00–4.00), confirming substantial baseline comorbidity burden and elevated mortality risk in our patient population. Hypertension (50.0%) and diabetes mellitus (33.3%) were the most common comorbidities in our series, with only two patients having no associated comorbidities. The clinical presentation of GI has no distinctive form. Intermittent abdominal pain and vomiting resulting from stone migration are common features, and obstruction signs vary depending on the impaction site. Similarly, in our study, all patients presented with abdominal pain (100%), vomiting was frequent (91.7%), while fever was noted in only one case (8.3%). The mean time from onset of the symptoms to hospital admission was 3.83 ± 2.08 days, consistent with the median delay of 3 days reported by Ayantunde et al. [5]. Laboratory analysis upon admission revealed elevated inflammatory markers consistent with acute obstruction and systemic inflammation.

Imaging serves as the primary diagnostic modality for GI. Rigler’s triad, consisting of pneumobilia, intestinal obstruction, and ectopic gallstone, is pathognomonic of GI; however, it is an inconstant finding, appearing in less than 50% of plain abdominal X-ray [11,12]. Contrast-enhanced CT has become the gold standard for diagnosing GI, with sensitivity (93%), specificity (100%), and accuracy (99%) as reported by Yu et al. [13]. CT is crucial for the identification of the obstruction site, the presence of bilioenteric fistula, and potential complications such as bowel ischemia or perforation.

In our study, all 12 patients underwent CT, and Rigler’s triad was identified in 91.7% of patients, a rate substantially higher than the traditionally reported rates, reflecting CT’s superior sensitivity compared to X-ray. Small bowel dilatation exceeding 3 cm and air-fluid levels were seen in 91.7% of patients (n = 11/12); one patient with duodenal stone impaction did not show an air-fluid level. In our series, 66.7% of stones were located in the distal ileum, consistent with the literature. Only one patient had a stone impacted in the duodenum, representing Bouveret’s syndrome, which accounts for less than 10% of GI cases [4]. None of our patients underwent preoperative USG, which aligns with current practice favoring CT in emergency presentations.

While surgical intervention remains the cornerstone of treatment for GI, alternative management strategies have been explored. Conservative management with observation and supportive care, particularly in high-risk patients with significant comorbidities, has been reported in selected cases with small stones (<2–2.5 cm) regardless of location, though it carries risks of prolonged obstruction and perforation [14,15,16]. Clavien et al. reported spontaneous passage with conservative treatment in selected patients [17]. Endoscopic intervention is feasible for stones accessible via endoscopy: proximal small bowel obstructions (Bouveret’s syndrome, duodenal impaction) and colonic obstructions. For colonic gallstone ileus, Augustin et al. recommend endoscopic approaches as the first-line option for stones > 2 cm. The most commonly used endoscopic technique is mechanical lithotripsy, with success rates of approximately 40% for both Bouveret’s syndrome and colonic gallstones [16,18,19]. Other options include extracorporeal shockwave lithotripsy, electrohydraulic lithotripsy, and laser lithotripsy (Nd:YAG), with varying degrees of success rates ranging from 9% to 40% [19,20,21,22,23,24]. Endoscopic options are limited by large stone size, difficulty in fragmenting hard stones, and the risk of stone fragments causing secondary obstruction. When endoscopic approaches fail or are contraindicated, surgical intervention becomes necessary. In contrast, for distal small bowel obstructions (jejunum and ileum), which constituted the majority of our cases, these endoscopic modalities are not feasible because of the location of the impaction.

In our series, none of the patients underwent conservative or endoscopic management. The mean stone size of 3.19 ± 0.6 cm exceeded the threshold for conservative management. Additionally, 66.7% of stones were in the distal ileum, making endoscopic intervention impossible. Although one patient had Bouveret’s syndrome, endoscopic extraction was not considered due to the large stone size (5 cm), which limits lithotripsy success. Consequently, surgical management was indicated in all cases, consistent with the literature emphasizing surgical approach as the definitive treatment [2].

There is an ongoing debate over the optimal surgical approach for GI, with treatment strategies ranging from only enterolithotomy to one-stage or two-stage procedures including cholecystectomy and fistula repair. The mainstay of the management is urgent surgical decompression, as metabolic derangements are commonly present at initial presentation. Enterolithotomy alone is the most commonly performed procedure worldwide [2,5] and was employed in 66.7% (n = 8) of our patients. Enterolithotomy is technically straightforward, requires shorter operative time (mean 76.4 ± 9.2 min overall; open: 72.5 ± 7.1 min, n = 6; laparoscopic: 88 ± 2.8 min, n = 2 in our series), and minimizes surgical trauma. However, enterolithotomy alone leaves the biliary fistula unaddressed with a 5% recurrence risk [11]. In our study, one patient (8.3%) who underwent laparoscopy-assisted enterolithotomy experienced recurrence one year postoperatively, requiring a one-stage procedure at another institution.

One-stage procedures were performed in 33.3% (n = 4) of our patients. This approach has its benefits, as since it may prevent future cholecystitis, cholangitis, malabsorption due to fistula formation, and gallbladder carcinoma [25]. However, one-stage surgery requires longer operative time and may carry higher morbidity in high-risk patients. In our study, all patients undergoing one-stage procedures had residual gallstones in the gallbladder on preoperative CT (4/4 vs. 3/8 in enterolithotomy-only patients). Two-stage surgery involving initial enterolithotomy followed by delayed cholecystectomy and fistula repair was not performed in any of our patients, though it may be appropriate for patients who require definitive biliary treatment and are too unstable for one-stage procedures.

The role of laparoscopy in GI management has increased over the years. Recent studies, case reports, and small series continue to demonstrate the feasibility of laparoscopic approaches in selected patients [26,27]. Dreifuss et al. reported successful total laparoscopic resolution of GI with favorable outcomes [28]. Ferretti et al. described laparoscopic management of sigmoid colon GI, demonstrating feasibility even in atypical locations [29]. Vallejo et al. reported successful laparoscopic-assisted enterolithotomy for recurrent GI [30]. Jakubauska M. et al. reported a successful laparoscopic approach in 3 of their cases [31]. Laparoscopic approaches were successfully employed in 41.7% (n = 5) of cases in our series, including two enterolithotomy cases alone and three one-stage procedures with no conversion to open. This success rate was achieved with experienced surgeons at our tertiary center, demonstrating that minimally invasive approaches are feasible with appropriate patient selection and surgical expertise.

Advanced age, comorbidities, delayed presentation, and the complexity of the surgery contribute to the high rate of postoperative complications in GI patients. In our series, complications were observed in 41.7% of patients, with two patients (16.7%) requiring intervention (Clavien-Dindo Grade IIIa), and one patient (8.3%) who died from aspiration pneumonia on postoperative day 4 following open enterolithotomy. Our mortality rate of 8.3% is consistent with recent large series reporting 5.5–7% mortality [32] and lower than the 22.7% reported by Ayantunde et al. [5]. Published data and our experience highlight that deaths occur due to patients’ comorbidities rather than the surgical techniques. The median (IQR) hospital stay was 6.00 (5.25–7.75) days and was comparable to reported literature. Four patients (33.3%) required ICU admission postoperatively, with a mean ICU stay of 1.33 ± 2.57 days. This reflects the high-risk profile of our cohort and the potential for serious postoperative complications in elderly GI patients.

Based on our experience and literature review, we suggest the following considerations for GI management. In hemodynamically stable elderly patients with significant comorbidities (ASA III-IV), enterolithotomy alone may be a reasonable first-line strategy, potentially accepting a minor recurrence risk (less than 5%) in exchange for reduced operative burden and mortality. For younger, healthier patients (ASA I–II) with persistent gallstones in the gallbladder, definitive one-stage surgery may be considered. However, our small cohort size precludes the development of definitive treatment algorithms, and decisions should be individualized based on institutional expertise and patient-specific factors.

Our study has several limitations. First, the small sample size (n = 12) and retrospective design limit statistical power and the ability to determine the best surgical approach. Second, treatment allocation was not randomized but based on ASA classification and clinical judgment. All ASA IV patients underwent enterolithotomy alone by open approach, while laparoscopy was only used in ASA II-III patients. This introduces selection bias, precluding direct statistical comparison of outcomes between surgical approaches. Third, our laparoscopic findings may not be widely generalizable. Our success rate was achieved with experienced surgeons at our tertiary center. Centers without similar laparoscopic expertise may not be able to reproduce these results. Despite these limitations, our study has notable strengths. Due to the rarity of GI, large-scale prospective studies are rarely feasible, and most published series report cohorts of similar or smaller size. Our relatively long follow-up period, with a median of 33.00 months, IQR 7.00–66.00, allows for assessment of late complications and recurrence.

## 5. Conclusions

Gallstone ileus remains a rare but serious complication of cholelithiasis in elderly patients, posing diagnostic challenges. Management should be individualized, as surgery remains the cornerstone of treatment. In our retrospective series, enterolithotomy alone was commonly performed in high-risk patients, while one-stage procedures were performed in stable patients with residual gallstones. Our findings suggest that laparoscopic approaches are feasible in carefully selected hemodynamically stable patients with appropriate surgical expertise, though larger studies are needed to define optimal patient selection criteria and comparative outcomes.

## Figures and Tables

**Table 1:** Demographic and Clinical features.

Variable	Total (n = 12)
Age (years), mean ± SD	70.25 ± 10.20
Sex, n (%)	
Female	10/12 (83.33)
Male	2/12 (16.67)
ASA score, n (%)	
ASA II	3/12 (25.00)
ASA III	6/12 (50.00)
ASA IV	3/12 (25.00)
Charlson Comorbidity Index, median (IQR) [range]	3.00 (2.00–4.00) [0–6.00]
Comorbidities, n (%)	
Hypertension	9/12 (75.00)
Diabetes mellitus	4/12 (33.33)
Hyperlipidemia	1/12 (8.33)
Atrial fibrillation	1/12 (8.33)
Chronic renal failure	1/12 (8.33)
Rheumatoid arthritis	1/12 (8.33)
Hypothyroidism	1/12 (8.33)
None	2/12 (16.67)
Symptoms, n (%)	
Abdominal pain	12/12 (100)
Vomiting	11/12 (91.67)
Constipation	2/12 (16.67)
History of acute cholecystitis, n (%)	None
History of cholelithiasis, n (%)	2/12 (16.67)
Time before admission (days), mean ± SD	3.83 ± 2.08
Laboratory analysis,	
Wbc (10^3^/µL), mean ± SD	13.99 ± 4.56
Hb (g/dL), mean ± SD	13.29 ± 1.74
Crp (mg/L), mean ± SD	73.58 ± 80.57
Ast (IU/L), mean ± SD	34.23 ± 18.06
Alt (IU/L), mean ± SD	37.57 ± 17.58
Ggt (U/L), median (IQR) [range]	54.50 (26.4–106.0) [18.2–158]
Ldh (U/L), median (IQR) [range]	199.50 (160.0–227.5) [140–307]
T.bil (mg/dL), median (IQR) [range]	0.92 (0.74–1.10) [0.47–1.5]
D.bil (mg/dL), median (IQR) [range]	0.38 (0.305–0.47) [0.22–0.76]
Ind bil (mg/dL), mean ± SD	0.52 ± 0.20
Urea (mg/dL), mean ± SD	49.17 ± 42.55
Creatinine (mg/dL), median (IQR) [range]	0.90 (0.87–1.38) [0.71–1.83]
K (mEq/L), mean ± SD	4.12 ± 0.42
Ca (mg/dL), mean ± SD	8.85 ± 0.65
Na (mEq/L), mean ± SD	136.42 ± 3.45

SD (stanard deviation), Wbc (white blood cell), Hb (hemoglobin), Crp (C-reactive protein), Ast (aspartate aminotransferase), Alt (alanine aminotransferase), Ggt (gamma-glutamyl transferase), Ldh (lactate dehydrogenase), T.bil (total bilirubin), D.bil (direct bilirubin), Ind bil (Indirect bilirubin), K (potassium), Ca (calcium), Na (sodium).

**Table 2:** Imaging and preoperative findings.

Preoperative plain X-ray, n (%)	12/12 (100)
Air fluid level	11/12 (91.67)
Pneumobilia	2/12 (16.67)
No finding	1/12 (8.33)
Preoperative CT, n (%)	12/12 (100)
- Air fluid level	11/12 (91.67)
- Rigler’s triad	11/12 (91.67)
- Small bowel dilatation > 3 cm	11/12 (91.67)
- Free fluid in the abdominal cavity	4/12 (33.33)
- Mean stone size cm, mean ± SD (range)	3.19 ± 0.62
- Mean number of stones, mean ± SD (range)	1.6 ± 1.2
Stone impaction site, n (%)	
- distal ileum	8/12 (66.67)
- proximal ileum	2/12 (16.67)
- jejunum	1/12 (8.33)
- duodenum	1/12 (8.33)
Previous operations, n (%)	
None	7/12 (58.33)
Hysterectomy	1/12 (8.33)
Cesarean section	1/12 (8.33)
Peptic ulcer perforation	1/12 (8.33)
Umblical hernia	1/12 (8.33)

**Table 3:** Surgical details and outcomes.

Operation type, n (%)	
Enterolithotomy	8/12 (66.67)
Open	6/8 (75.00)
Laparoscopic	2/8 (25.00)
One-stage procedure	4/12 (33.33)
Open	1/4 (25.00)
Laparoscopic	3/4 (75.00)
Operation time (min), mean ± SD	
Enterolithotomy	76.82 ± 9.69
Open	72.50 ± 6.65
Laparoscopic	88.00 ± 2.82
One-stage procedure	150.00 ± 27.57
Open	115.00
Laparoscopic	161.00 ± 18.01
Site of bilioenteric fistula, n (%)	Bilioduodenal 12/12 (100)
Number of stones, mean ± SD (range)	1.67 ± 1.15
Stone size (cm), mean ± SD (range)	3.19 ± 0.62
Hospital stay (days), median (IQR) [range]	6.00 (5.25–7.75) [4–12]
ICU admission, n (%)	4/12 (33.33)
ICU stay (days), mean ± SD	1.33 ± 2.57
Clavien-Dindo complication, n (%)	
Grade 0	7/12 (58.3)
Grade I	2/12 (16.7)
Grade IIIa	2/12 (16.7)
Grade V	1/12 (8.3)
Follow-up (months), median (IQR) [range]	33.00 (7.00–66.00) [0.10−120.00]
- Recurrence, n (%)	1/12 (8.3)
- Mortality, n (%)	1/12 (8.3)

## Data Availability

The datasets generated and/or analyzed during the current study are available from the corresponding author upon reasonable request.
